# Structure of the TXNL1-bound proteasome

**DOI:** 10.1038/s41594-025-01639-w

**Published:** 2025-08-06

**Authors:** Jingjing Gao, Christopher Nardone, Matthew C. J. Yip, Haruka Chino, Xin Gu, Zachary Mirman, Michael J. Rale, Joao A. Paulo, Stephen J. Elledge, Sichen Shao

**Affiliations:** 1 Department of Cell Biology, Harvard Medical School, Boston, MA USA; 2 Department of Genetics, Harvard Medical School, Boston, MA USA; 3 Division of Genetics, Department of Medicine, Brigham and Women’s Hospital, Boston, MA USA; 4 Department of Neurobiology, Harvard Medical School, Boston, MA USA; 5 Howard Hughes Medical Institute, Boston, MA USA

**Keywords:** Cryoelectron microscopy, Proteasome, Proteasome

## Abstract

Proteasomes degrade diverse proteins in different cellular contexts through incompletely defined regulatory mechanisms. Here we report the cryo-EM structure of human thioredoxin-like protein 1 (TXNL1) bound to the 19S regulatory particle of proteasomes via interactions with PSMD1 (Rpn2), PSMD4 (Rpn10) and PSMD14 (Rpn11). Proteasome binding is necessary for the ubiquitin-independent degradation of TXNL1 upon cellular exposure to metal- or metalloid-containing oxidative agents, thereby establishing a structural requirement for the stress-induced degradation of TXNL1.

## Abstract (web summary)

Gao, Nardone et al. report the cryo-EM structure of human TXNL1 bound to the proteasome and reveal interactions required for the stress-induced degradation of TXNL1, an abundant protein that may regulate proteasomal activity.

## Main

Proteasomes are the primary protein degradation machinery in eukaryotes and comprise a 20S core particle harboring proteases capped by one or two 19S regulatory particles (RPs)^1^. Conventionally, proteasomes degrade poly-ubiquitylated proteins that are recruited by ubiquitin receptors on the 19S RP: PSMD2 (Rpn1), PSMD4 (Rpn10) and ADRM1 (Rpn13)^2–5^. These substrates are then unfolded by translocating through the AAA-ATPase motor at the base of the 19S RP before entering the core particle for proteolysis^1,6,7^. Successful proteasomal degradation also involves PSMD14 (Rpn11), an essential zinc metalloprotease on the 19S RP that deubiquitylates substrates to promote their efficient translocation^8–10^.

Some proteins can undergo proteasomal degradation without a requirement for ubiquitylation^11–15^. For example, the proteasomal adapter midnolin directly captures specific nuclear proteins to promote their degradation^12,13^. It is unclear whether and how other proteins engage proteasomes for ubiquitin-independent degradation. In this study, we report the cryo-EM structure of the human proteasome bound to thioredoxin-like protein 1 (TXNL1), a conserved thioreductase that interacts stably with proteasomes and, in *Schizosaccharomyces pombe*, shows genetic interactions with proteasome regulators^16–18^. Our structure reveals key binding interfaces between TXNL1 and proteasomal subunits required for the ubiquitin-independent degradation of TXNL1 upon cellular exposure to certain compounds that cause oxidative stress. Our findings identify the molecular interactions required for the stress-induced degradation of an abundant protein that may regulate proteasomal activity^18^.

## Results and discussion

### Cryo-EM structure of the TXNL1-bound proteasome

**Fig. 1:**
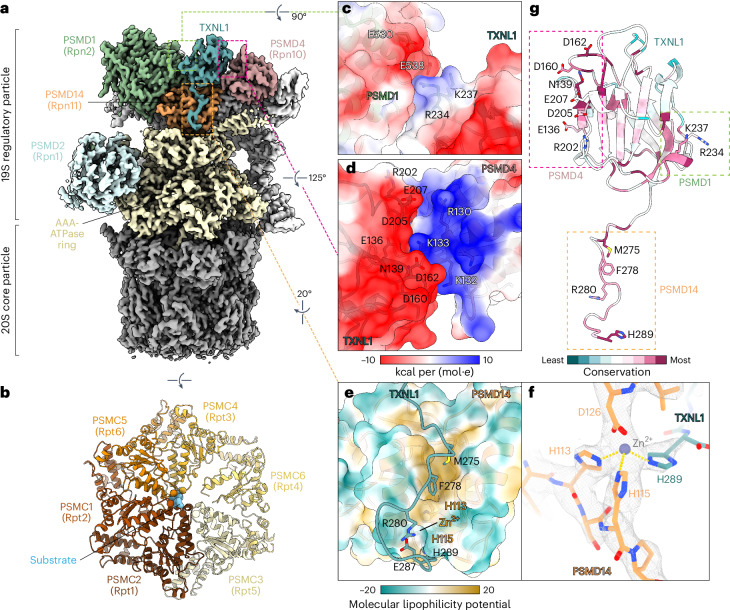
Structure of TXNL1 bound to the proteasome. **a**, Cryo-EM map of TXNL1 on the proteasome. **b**, Model of the AAA-ATPase ring with density corresponding to a translocating substrate (light blue), viewed from above the substrate entry site. **c**,**d**, TXNL1 binding to PSMD1 (**c**) and PSMD14 (**d**) occurs through electrostatic interactions. **e**, The C-terminal tail of TXNL1 interacts with PSMD14 through hydrophobic interactions. **f**, H289 of TXNL1 coordinates the catalytic zinc ion associated with PSMD14. **g**, Proteasome-interacting domain of TXNL1 colored by conservation with residues at proteasomal interfaces labeled.

**Extended Data Fig. 1:**
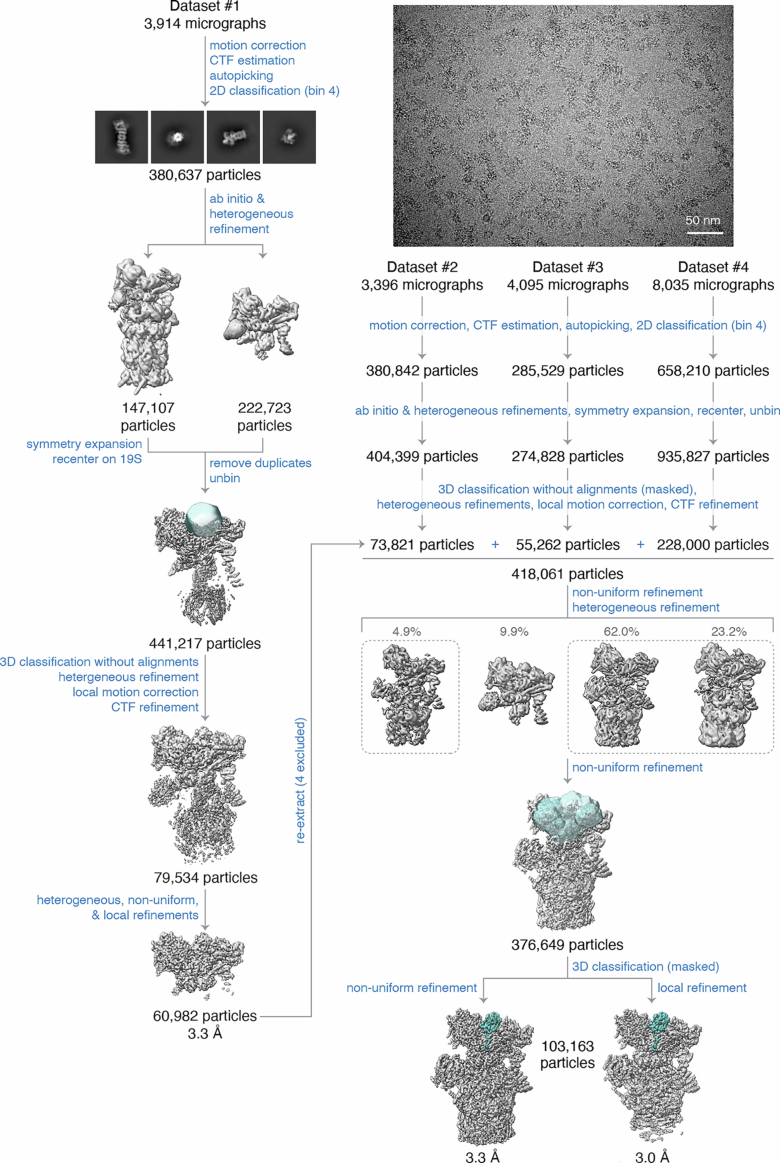
Cryo-EM data processing. Summary of cryo-EM data processing and classification strategy showing a representative micrograph, 2D class images, and 3D reconstructions.

**Extended Data Fig. 2:**
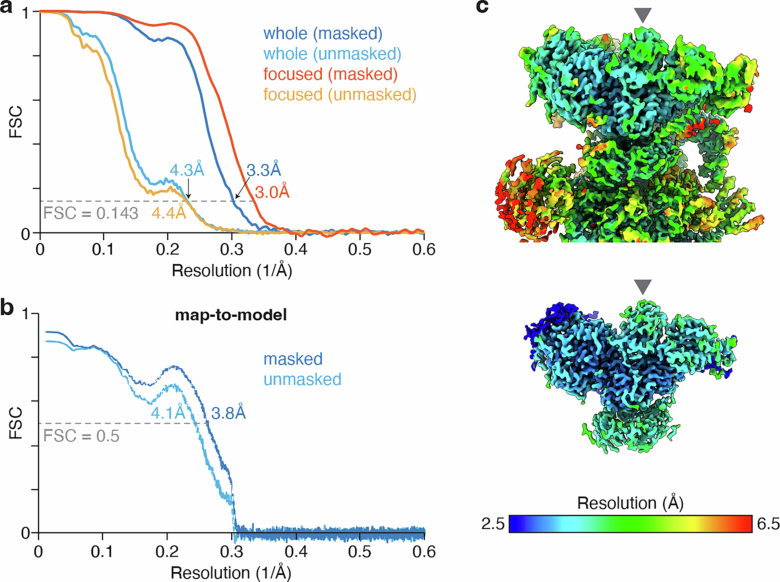
Cryo-EM map and model quality. **a**, Fourier shell correlation (FSC) vs. resolution (1/Å) curves for the indicated cryo-EM maps. **b**, The whole (top) and focused (bottom) cryo-EM map colored by local resolution. Arrow denotes TXNL1. **c**, Model vs. map FSC curves.

We determined the structure of the TXNL1-bound proteasome to an overall resolution of 3.0–3.3 Å from cryo-EM datasets of affinity-purified midnolin–proteasome complexes (Fig. 1a, Table 1 and Extended Data Figs. 1 and 2). In addition to revealing the structures of midnolin-bound proteasomes described elsewhere^19^, three-dimensional (3D) classifications revealed a subset of particles contributing to reconstructions of the proteasome or 19S RP with a well-resolved density that has not been previously reported (Fig. 1a and Extended Data Figs. 1 and 2). We identified this protein as TXNL1 based on amino acid sequences assigned to the density by ModelAngelo^20^.

**Table 1:** Cryo-EM data collection, refinement and validation statistics

	TXNL1 + proteasome PDB 9BW4, EMD-44952	TXNL1 + proteasome (local refinement) EMD-44949
**Data collection and processing**		
Magnification	105,000	105,000
Voltage (kV)	300	300
Electron exposure (*e*^−^/Å^2^)	50 to 54	50 to 54
Defocus range (μm)	−0.8 to −2.2	−0.8 to −2.2
Pixel size (Å)	0.825	0.825
Symmetry imposed	*C*_1_	*C*_1_
Initial particle images (no.)	1,765,798	1,765,798
Final particle images (no.)	103,163	103,163
Map resolution (Å)	3.3	3.0
FSC threshold	0.143	0.143
Map resolution range (Å)	2.9 to 51.6	2.5 to 25.0
**Refinement**		
Initial model used (PDB code)	AlphaFold2, ModelAngelo	
Model resolution (Å)	3.8	
FSC threshold	0.5	
Model resolution range (Å)	3.8 to 82.8	
Map sharpening *B* factor (Å^2^)	−55.6	
Model composition		
Non-hydrogen atoms	90,418	
Protein residues	11,464	
Ligands	1 Zn^2+^	
	5 Mg^2+^	
	3 ATP	
	3 ADP	
*B* factors (Å^2^)		
Protein	80.37	
Ligand	75.31	
R.m.s. deviations		
Bond lengths (Å)	0.006	
Bond angles (°)	1.033	
**Validation**		
MolProbity score	2.33	
Clashscore	17.92	
Poor rotamers (%)	0.03	
Ramachandran plot		
Favored (%)	88.86	
Allowed (%)	11.06	
Disallowed (%)	0.08	

**Extended Data Fig. 3:**
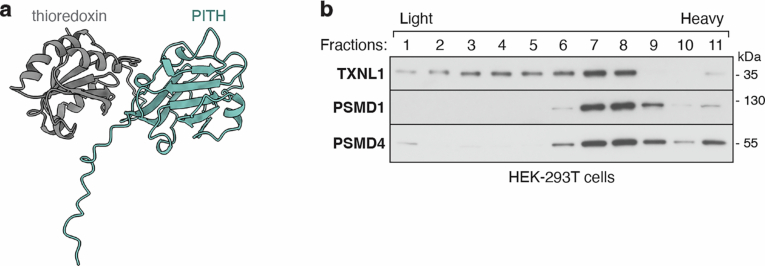
Characteristics of TXNL1. **a**, AlphaFold prediction of TXNL1 showing the N-terminal thioredoxin domain and C-terminal proteasome-interacting thioredoxin (PITH) domain. **b**, A substantial portion of TXNL1 co-migrates with proteasomal components in size fractionations of HEK-293T cell lysates by sucrose gradient ultracentrifugation, assayed by SDS-PAGE and immunoblotting, representative of 2 independent experiments. Source data

TXNL1 is an abundant (>1 µM)^21^, 32-kDa protein with an N-terminal thioredoxin domain and a C-terminal proteasome-interacting thioredoxin (PITH) domain^16–18^ (Extended Data Fig. 3a). In addition, native size fractionations revealed that a substantial proportion of endogenous TXNL1 co-migrated with proteasomes (Extended Data Fig. 3b). A high proteasomal occupancy may explain the presence of TXNL1-bound proteasomes in our cryo-EM datasets.

**Extended Data Fig. 4:**
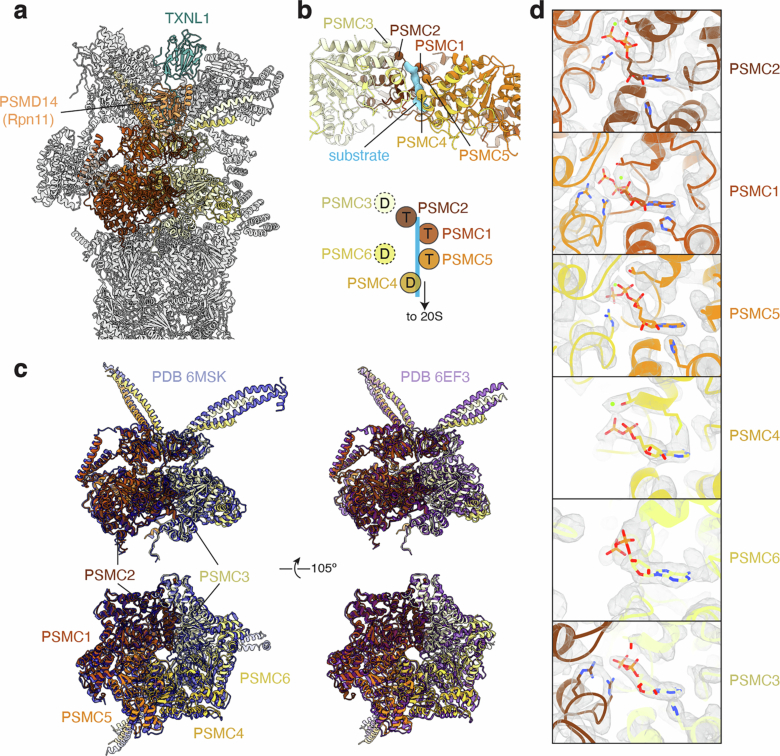
Characteristics of the TXNL1-bound proteasome. **a**, Structure of the TXNL1-bound proteasome with TXNL1, PSMD14, and AAA-ATPase subunits colored as in Fig. 1b**b**, Clipped side view of the AAA-ATPase ring (top), colored by subunit, with the position of aromatic pore loop residues shown in spheres and substrate density in blue. Scheme (bottom) depicting the configuration and nucleotide (T – ATP, D – ADP)-bound states of the ATPase subunits relative to the substrate (blue). **c**, Superposition of the AAA-ATPase ring with that in the E_D2_ state of the human proteasome (PDB 6MSK; transparent dark blue; middle) or the 4D state of the yeast proteasome (PDB 6EF3; transparent dark purple, right). **d**, Map density (mesh) and model of the nucleotide binding site in the indicated AAA-ATPase subunit (from top to bottom: PSMC2, PSMC1, PSMC5, PSMC4, PSMC6, and PSMC3).

Although the thioredoxin domain of TXNL1 is not resolved in our map, we could model the entire PITH domain (residues 118–289) bound to PSMD1, a structural component of the 19S RP, the ubiquitin receptor PSMD4 and the deubiquitinase PSMD14. Below the TXNL1 binding site, the AAA-ATPase ring is in a state of active translocation, with a clear substrate polypeptide density that is likely an averaged mixture of endogenous proteasomal substrates and visible nucleotide density associated with each subunit (Fig. 1b and Extended Data Fig. 4a–d). The conformation of the AAA-ATPase is most similar to the human E_D2_ (root mean square (r.m.s.) deviation = 3.0 Å) or yeast 4D (r.m.s. deviation = 2.0 Å) proteasomal states^6,7^, with PSMC3 (Rpt5) at the top of the spiral AAA-ATPase configuration and PSMC6 (Rpt4) fully disengaged from the substrate (Extended Data Fig. 4b–d).

### TXNL1 interactions with proteasomal components

Electrostatic interactions facilitate TXNL1 binding to PSMD1 and PSMD4 (Fig. 1c,d). Residues involved in these interactions include R234 of TXNL1, which interacts with an acidic patch on PSMD1 (Fig. 1c), and E136 and D162 of TXNL1, which interact with a basic interface on PSMD4 (Fig. 1d). The C-terminal unstructured tail of TXNL1 engages a hydrophobic groove on PSMD14 (Fig. 1e) and extends into the PSMD14 active site, where H289, the C-terminal residue of TXNL1, coordinates zinc along with H113 and H115 of PSMD14 (Fig. 1f,g). These interactions are consistent with another structural study of TXNL1-bound proteasomes^22^, and the amino acids on TXNL1 that interact with proteasomal subunits, particularly H289, are highly conserved (Fig. 1g).

**Fig. 2:**
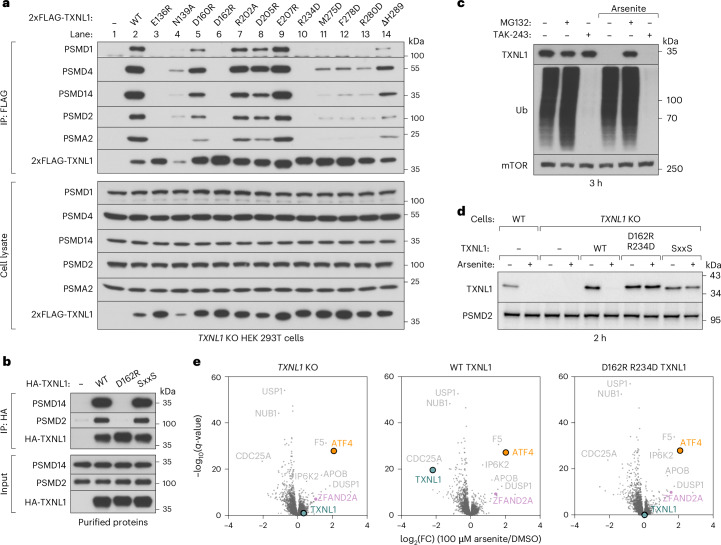
Proteasome binding is required for the stress-induced degradation of TXNL1. **a**, Immunoblotting of anti-FLAG IP (top) and input lysates (bottom) of *TXNL1* KO HEK 293T cells transfected with 2xFLAG-tagged TXNL1 variants, representative of three independent experiments. **b**, Immunoblotting of purified HA-tagged TXNL1 variants immunoprecipitated in the presence of purified proteasomes, representative of three independent experiments. **c**, TXNL1 degradation upon arsenite treatment is proteasome-dependent but ubiquitin-independent. Immunoblotting of HEK 293T cells treated without or with 100 µM arsenite, 10 µM MG132, a proteasome inhibitor or 1 µM TAK-243, an inhibitor of the E1 ubiquitin-activating enzyme, representative of three independent experiments. **d**, TXNL1 degradation upon arsenite exposure requires proteasome binding and thioreductase activity. Immunoblotting from WT or *TXNL1* KO HEK 293T cells stably complemented without or with WT and mutant versions of TXNL1 by lentiviral transduction, representative of three independent experiments. **e**, Volcano plots of showing the fold-change (log_2_(FC)) of protein levels upon treatment with arsenite for 3 h of *TXNL1* KO HEK 293T cells complemented without or with WT or D162R R234D TXNL1. mTOR, mammalian target of rapamycin; Ub, ubiquitin. Source data

To validate the interaction interfaces observed in our structure, we performed coimmunoprecipitations from *TXNL1* knockout (KO) human embryonic kidney (HEK) 293T cells complemented with epitope-tagged TXNL1 variants (Fig. 2a). Wild-type (WT) TXNL1 associated with proteasomes, but the interaction was severely impaired by mutations to disrupt the interface with PSMD1 (R234D; lane 10), PSMD4 (E136R and D162R; lanes 3–6) or PSMD14 (lanes 11–14). With the exception of N139A TXNL1 (lane 4), we observed an inverse correlation between TXNL1 abundance and its ability to bind proteasomes, consistent with the previous observation that TXNL1 levels are increased upon proteasome inhibition^16^.

**Extended Data Fig. 5:**
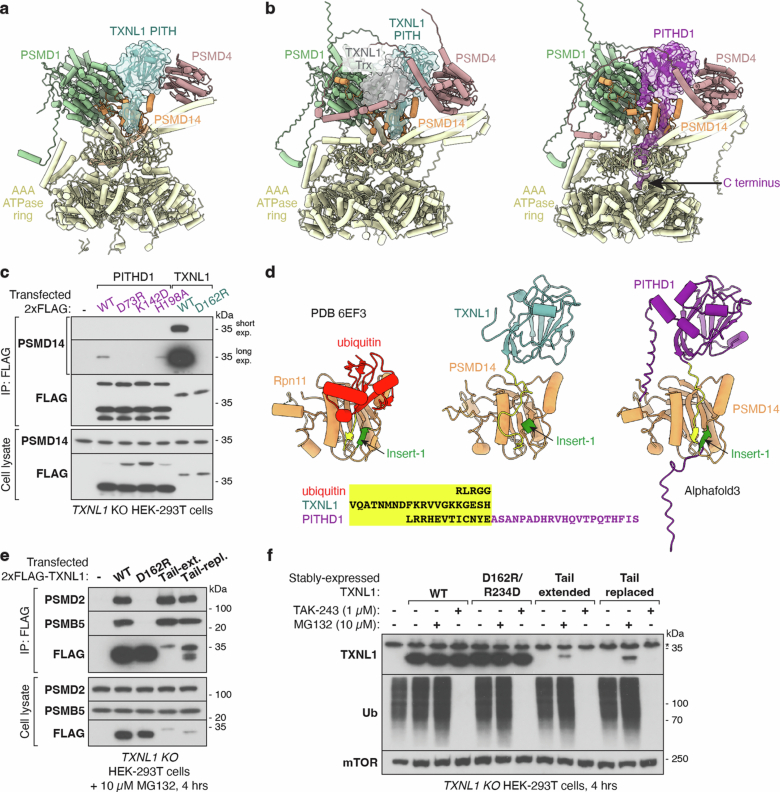
Common PITH domain interaction interfaces. **a**, Cryo-EM structure showing TXNL1 PITH domain interactions with the proteasome. **b**, Alphafold3 models of TXNL1 (left) or PITHD1 (right) with PSMD1, PSMD4, PSMD14, and the AAA-ATPase subunits show shared interaction interfaces. Note: PITHD1 has an extended C-terminal tail that may enter the central pore of the AAA-ATPase ring. **c**, Immunoblotting of anti-FLAG immunoprecipitates (IP, top) and input lysates (bottom) of *TXNL1* knockout (KO) HEK-293T cells transfected with 2xFLAG-tagged PITHD1 and TXNL1 variants, representative of 2 independent experiments. WT, wild type. D73 and K142 of PITHD1 are equivalent to D162 and R234 of TXNL1, respectively. **d**, Comparison of ubiquitin (red, left, PDB 6EF3), TXNL1 (teal, middle), and PITHD1 (purple, right, Alphafold3 model) engagement with PSMD14/Rpn11. The Insert-1 region of PSMD14/Rpn11 that assumes a β-hairpin when bound to ubiquitin is colored green. The C-terminal sequences of each factor that engage PSDM14/Rpn11 are highlighted in yellow and shown below. Note: ubiquitin and PITHD1, but not TXNL1, induce the β-hairpin Insert-1 conformation, and PITHD1 contains a C-terminal extension (purple) past the segment predicted to bind PSMD14. **e**, Same assay as in c, but from cells expressing TXNL1 variants in which the C-terminal tail was either extended (Tail-ext.) or replaced (Tail-repl.) with the PITHD1 C-terminal tail, as aligned in d, representative of 2 independent experiments. Cells were pre-treated with 10 µM MG-132 for 4 hrs. **f**, Immunoblotting of lysates of *TXNL1* KO cells that were transduced with lentivirus to stably express wild-type or mutant variants of TXNL1 and pre-treated with the indicated drugs for 4 hrs, representative of 2 independent experiments. *, nonspecific band. Source data

These interfaces also match AlphaFold3 predictions of 19S RP subunits with TXNL1 and PITHD1, which has a PITH domain but no thioreductase activity (Extended Data Fig. 5a,b). Although PITHD1 isolated proteasomes less efficiently than TXNL1, immunoprecipitations confirmed that mutations in the conserved interaction interfaces reduced proteasome binding to below detection in this assay (Extended Data Fig. 5c). In addition, when bound to TXNL1, the ubiquitin-interacting Insert-1 region of PSMD14 does not assume the β-hairpin conformation used to position ubiquitin for cleavage^6,7,23^ (Extended Data Fig. 5d). PITHD1 also has an extended C-terminal tail that is predicted to engage PSMD14 with a β-hairpin Insert-1 region and then enter the central pore of the AAA-ATPase ring^24^ (Extended Data Fig. 5b,d). Interestingly, extending the C-terminal tail of TXNL1 past H289 or replacing the C-terminal tail of TXNL1 with that of PITHD1 was sufficient to destabilize the protein in a proteasome-dependent, but ubiquitin-independent, manner (Extended Data Fig. 5e,f) (discussed below).

**Extended Data Fig. 6:**
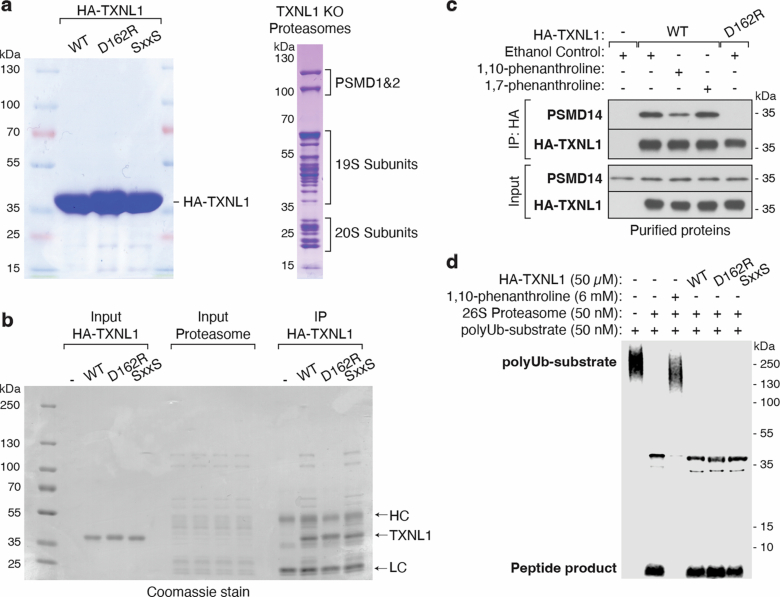
*In vitro* assays of TXNL1. **a**, SDS-PAGE and Coomassie staining of purified HA-tagged TXNL1 variants (left) and TXNL1-deficient human proteasomes (right), representative of 2 independent purifications. **b**, Coomassie stain of samples from Fig. 2b, representative of 3 independent experiments. IP, immunoprecipitation. **c**, Purified HA-TXNL1 variants were immunoprecipitated in the presence of purified proteasomes pre-incubated with 6 mM of the indicated compound for 30 min at room temperature before immunoblotting, representative of 2 independent experiments. **d**, Proteasomes were pre-incubated with TXNL1 or the PSMD14/Rpn11 inhibitor 1,10-phenanthroline for 30 min at room temperature before the addition of a polyubiquitylated (polyUb) sfGFP protein substrate for 10 min at 37 °C, representative of 2 independent experiments. Note: 1,10-phenanthroline inhibits deubiquitylation and degradation of the polyUb-substrate (lane 3), but an excess of TXNL1 does not (lane 4). Source data

Next, we validated TXNL1–proteasome interactions with purified components. Immunoprecipitations of purified HA-TXNL1 variants incubated with proteasomes from *TXNL1* KO HEK 293T cells confirmed that WT TXNL1 and a thioredoxin domain mutant (SxxS) isolated proteasomes, whereas D162R TXNL1, a proteasome binding-deficient mutant, did not (Fig. 2b and Extended Data Fig. 6a,b). TXNL1 binding to proteasomes was also impaired by preincubating proteasomes with 1,10-phenanthroline, a zinc chelator that inhibits PSMD14 deubiquitylation activity^8^, but not 1,7-phenanthroline, an inactive isomer (Extended Data Fig. 6c). This finding is consistent with our observation that H289 of TXNL1 coordinates the catalytic zinc of PSMD14 (Fig. 1f) and is important for proteasome engagement (Fig. 2a). However, unlike 1,10-phenanthroline, an excess of TXNL1 preincubated with purified proteasomes did not inhibit the in vitro deubiquitylation or degradation of a poly-ubiquitylated protein in an endpoint assay^25^ (Extended Data Fig. 6d).

### Arsenite-induced TXNL1 degradation requires proteasomal contacts

**Extended Data Fig. 7:**
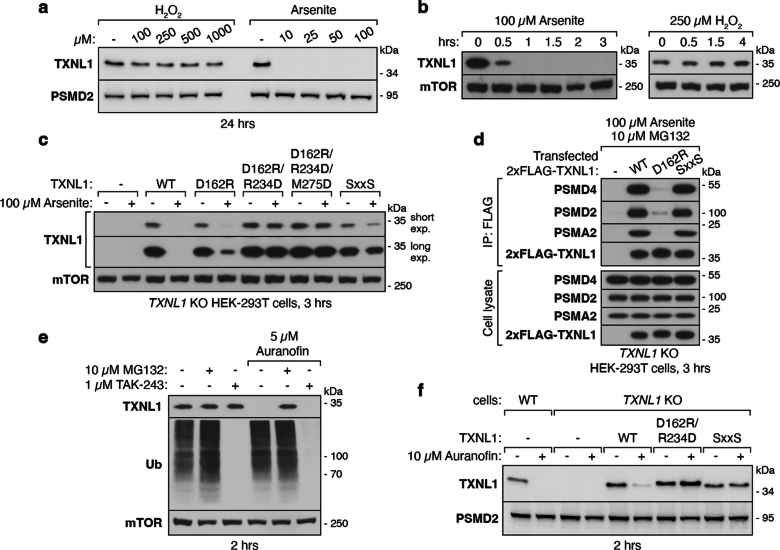
Requirements for stress-induced TXNL1 degradation. **a**, TXNL1 is rapidly degraded upon cellular exposure to arsenite but not hydrogen peroxide. Immunoblotting of HEK-293T cells treated with the indicated concentration of oxidative agents for 24 hrs, representative of 3 independent experiments. **b**, Immunoblotting of HEK-293T cells treated with 100 µM arsenite or 250 µM hydrogen peroxide over a time course, representative of 3 independent experiments. **c**, Immunoblotting from *TXNL1* knockout (KO) HEK-293T cells that were stably complemented with wild type (WT) and mutant versions of TXNL1 by lentiviral transduction, representative of 3 independent experiments. D162, R234, and M275 reside at the interaction interfaces with PSMD1, PSMD4, and PSMD14, respectively. SxxS mutates the catalytic cysteines in the thioredoxin domain of TXNL1. Note: D162R TXNL1 does not co-purify proteasomes (Fig. 2b) but only weakly impairs arsenite-induced degradation, indicating that it still has some affinity for proteasomes, likely through the interface with PSMD1 that is disrupted by the R234D mutation. **d**, Immunoblotting of anti-FLAG immunoprecipitates (IP, top) and input lysates (bottom) of *TXNL1* KO HEK-293T cells transfected with 2xFLAG-tagged TXNL1 variants and treated with 100 µM arsenite and 10 µM MG132 for 3 hrs, representative of 3 independent experiments. Note: WT and SxxS TXNL1 still interact with proteasomes in the presence of arsenite. **e**, TXNL1 degradation upon auranofin treatment requires proteasome binding and thioredoxin activity. Assay as in Fig. 2c but with auranofin treatment in place of arsenite treatment, representative of 3 independent experiments. **f**, Assay as in Fig. 2d but with auranofin treatment in place of arsenite treatment, representative of 3 independent experiments. Source data

It has been reported that exposing cells to arsenite, a chemotherapeutic used to treat acute promyelocytic leukemia that causes oxidative stress, reduces TXNL1 protein levels^17,26^. We confirmed that TXNL1 is rapidly destabilized in cells treated with arsenite, but not hydrogen peroxide (Extended Data Fig. 7a,b). The proteasome inhibitor MG132 abrogated the arsenite-triggered degradation of TXNL1, but TAK-243, an inhibitor of the E1 ubiquitin-activating enzyme^27^, did not (Fig. 2c). Complementing *TXNL1* KO cells with TXNL1 variants showed efficient arsenite-induced degradation of WT TXNL1, but not of D162R R234D TXNL1 deficient in proteasome binding (Fig. 2d and Extended Data Fig. 7c). The SxxS thioredoxin mutant of TXNL1 also was not destabilized, even though it could still bind proteasomes, in the presence of arsenite (Fig. 2d and Extended Data Fig. 7c,d). We observed similar results using auranofin, a gold-containing oxidizing agent^28–30^, in place of arsenite (Extended Data Fig. 7e,f). These observations indicate that metal- or metalloid-containing oxidative agents trigger a cellular response leading to ubiquitin-independent degradation of TXNL1 that requires both the proteasome binding activity and catalytic cysteines of TXNL1.

**Extended Data Fig. 8:**
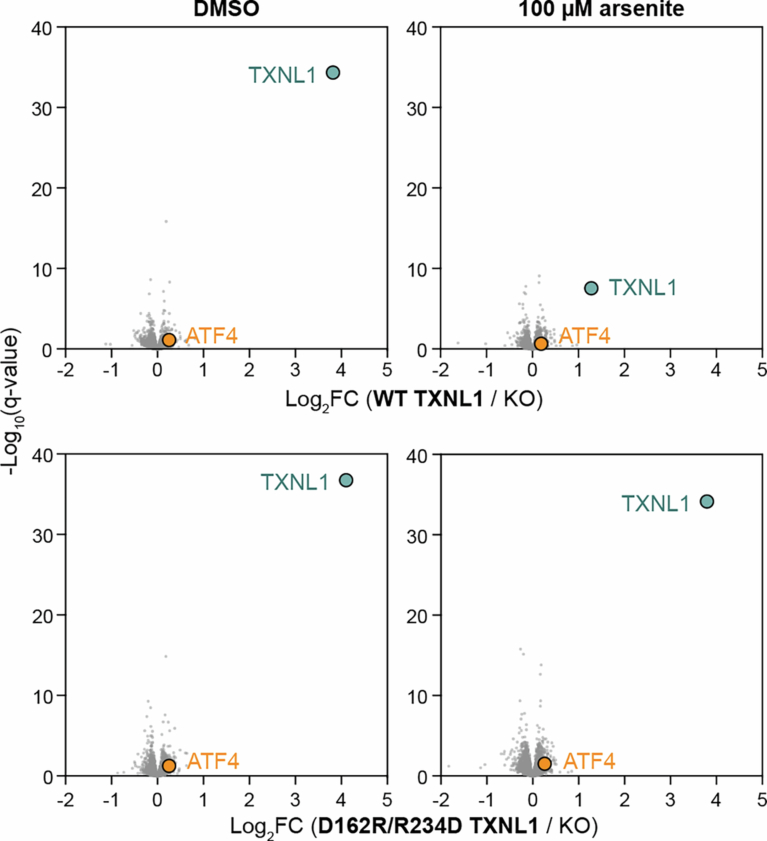
Proteome changes upon arsenite exposure with TXNL1 variants. Selective degradation of TXNL1 upon arsenite exposure requires proteasome binding. Volcano plots of multiplexed proteomics data showing the fold-change (Log_2_FC) of protein levels in *TXNL1* KO HEK-293T cells complemented with the indicated TXNL1 variant compared to KO cells without (left) and with (right) arsenite treatment. Two-sided Student’s t-test p-values were adjusted for multiple comparisons using the Benjamini and Hochberg method. Note: arsenite treatment selectively lowers the level of WT but not D162R/R234D TXNL1.

**Extended Data Fig. 9:**
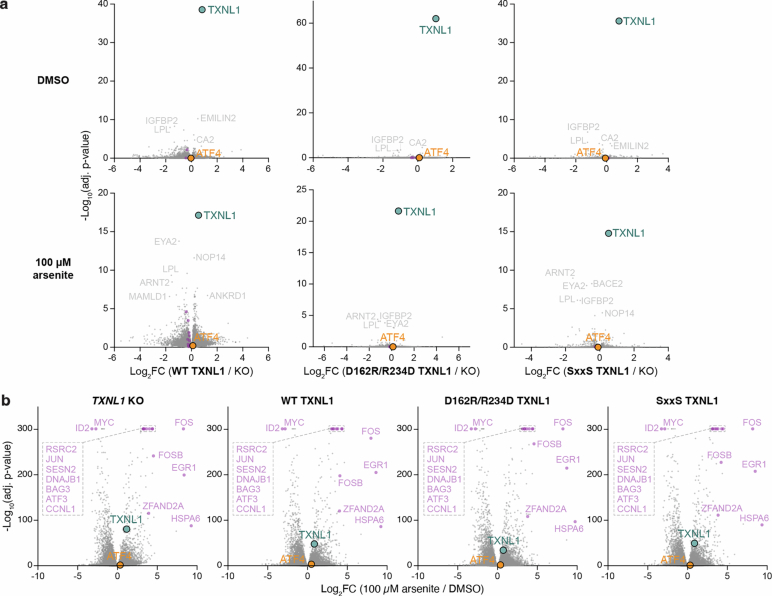
Transcriptional changes upon cellular arsenite exposure. **a**, Volcano plots of RNA sequencing data showing the fold-change (Log_2_FC) of transcript levels in *TXNL1* knockout (KO) HEK-293T cells complemented with wild type (WT) TXNL1, D162R/R234D TXNL1 deficient in proteasome binding, or SxxS TXNL1 deficient in thioreductase activity compared to KO cells without (top) or with (bottom) arsenite treatment. Two-sided Wald test p-values were adjusted for multiple comparisons using the Benjamini and Hochberg method. Note: TXNL1 is the main transcript that is significantly changed in both conditions. **b**, Volcano plots of RNA sequencing data as in **a** but showing the fold-change (Log_2_FC) of transcript levels in *TXNL1* KO cells without (left) or with complementation with WT TXNL1 or the indicated TXNL1 mutants upon arsenite treatment. Note: changes in stress-responsive transcript (purple) levels are not impacted by TXNL1 expression.

To further investigate this response and the general role of TXNL1, we used multiplexed proteomics to analyze *TXNL1* KO cells complemented without or with WT or D162R R234D TXNL1 (Fig. 2e, Extended Data Fig. 8 and Supplementary Table 1). We also performed RNA sequencing of the same cell lines as well as *TXNL1* KO cells complemented with SxxS TXNL1 (Extended Data Fig. 9 and Supplementary Table 2). These datasets revealed that re-expressing any TXNL1 variant in KO cells minimally impacted the proteome and transcriptome (Extended Data Figs. 8 and 9a). Supporting our immunoblotting results, arsenite treatment selectively destabilized WT, but not D162R R234D TXNL1 protein levels and induced a dramatic transcriptional response and post-transcriptional upregulation of the stress-induced transcription factor ATF4 in all cell lines (Fig. 2e and Extended Data Fig. 9b). However, we did not observe specific differences in the arsenite-dependent upregulation or downregulation of any protein or transcript other than TXNL1 across the cell lines. Thus, elucidation of the purpose and impact of TXNL1 degradation in cellular physiology remains to be determined.

### Implications

Altogether, our study reveals specific interactions required for proteasome binding and the stress-induced degradation of TXNL1. We hypothesize that arsenite may react with the thioredoxin domain of TXNL1 and induce conformational changes, such as unfolding or a tighter interaction with a proteasomal substrate, that initiates the translocation and degradation of TXNL1 in proximity to the AAA-ATPase. This idea may be supported by our observation that simply extending the C-terminal of tail of TXNL1 toward the AAA-ATPase ring is sufficient to induce constitutive ubiquitin-independent degradation of TXNL1 (Extended Data Fig. 5d,e).

In addition, based on the high abundance of TXNL1, its thioreductase activity and its position above the entry to the AAA-ATPase motor of actively translocating proteasomes, it is tempting to speculate that TXNL1 normally functions to reduce oxidized substrate proteins to facilitate their degradation. In this model, TXNL1 may cooperate with deubiquitylation by PSMD14 to promote efficient substrate translocation, which may involve TXNL1 coming on and off the proteasome when substrate-conjugated ubiquitins engage PSMD14 for removal. Because putative substrates that require TXNL1 for degradation are probably heterogeneous and constitute only a small fraction of proteins in a typical cell, like many proteins regulated by quality control pathways, identifying them may be challenging. Future insights, facilitated by the molecular interactions reported here, will require discovering specific substrates, cell types or cellular conditions that are particularly reliant on TXNL1.

## Methods

### Cryo-electron microscopy sample preparation, data collection, image processing and model building

Plasmid DNA encoding epitope-tagged midnolin variants was transiently transfected into HEK 293T cells for immunoprecipitation. The affinity purifications were partitioned by size-exclusion chromatography (SEC) and the fractions corresponding to proteasomes were collected. Then 3.5 µl of 4 mg ml^−1^ proteasomal complexes was applied to glow-discharged R0.6/1 UltrAuFoil 300 mesh grids (Quantifoil) and frozen in liquid ethane using a Vitrobot Mark IV (Thermo Fisher Scientific) set at 4 °C and 100% humidity with a 10 s wait time, 3 s blot time and +8 blot force.

Datasets were collected using a Titan Krios (Thermo Fisher Scientific) operating at 300 kV and equipped with a BioQuantum K3 imaging filter with a 20 eV slit width and a K3 summit direct electron detector (Gatan) in counting mode at a nominal magnification of ×105,000 corresponding to a calibrated pixel size of 0.825 Å. Semi-automated data collection was performed using SerialEM v.4.0.5 for four datasets with the following parameters. Dataset 1: 2.597-s exposures were fractionated into 50 frames, resulting in a total exposure of 52.98 *e*^−^/Å^2^. The defocus targets were −1.0 to −2.2 µm. Dataset 2: 2.652-s exposures were fractionated into 48 frames, resulting in a total exposure of 50.54 *e*^−^/Å^2^. The defocus targets were −1.0 to −2.2 µm. Dataset 3: 2.65-s exposures were fractionated into 50 frames, resulting in a total exposure of 53 *e*^−^/Å^2^. The defocus targets were −1.0 to −2.2 µm. Dataset 4: 3.02-s exposures were fractionated into 54 frames, resulting in a total exposure of 53.79 *e*^−^/Å^2^. The defocus targets were −0.8 to −1.8 µm.

Data processing was performed in cryoSPARC v.4.3.1. After patch-based motion correction and contrast transfer function (CTF) estimation, micrographs with severe contamination or poor CTF fits were removed. Then 3,914 (Dataset 1), 3,396 (Dataset 2), 4,095 (Dataset 3) or 8,035 (Dataset 4) micrographs were subjected to automated particle picking using templates generated from blob-based picking. The particles were extracted with a box size of 800 and downsampled to a box size of 200. After two-dimensional classification, 380,637 (Dataset 1), 380,842 (Dataset 2), 285,529 (Dataset 3) and 658,210 (Dataset 4) particles were selected for heterogeneous refinement using multiple reference volumes generated by ab initio reconstruction. Particles in the best classes were subjected to homogeneous refinement with *C*_2_ symmetry, followed by symmetry expansion. Afterwards, all particles were re-centered on the 19S particle, extracted with a box size of 440 and downsampled to a box size of 220. Good 19S particles after two-dimensional classification were then subjected to heterogeneous refinements and 3D classification without alignment using a mask over the TXNL1 binding site based on initial observation of density in this region from heterogeneous refinements of Dataset 1 (Extended Data Fig. 1).

In total, 418,061 particles from the four datasets in classes with density corresponding to the TXNL1 were unbinned, local motion corrected, combined and subjected to nonuniform and an additional round of heterogeneous refinement. Classes corresponding to intact proteasomes were selected and subjected to nonuniform refinement and 3D classification with a mask focused on TXNL1. The 103,163 particles with clear TXNL1 density were used for final nonuniform and local refinements using the same mask. TXNL1 was identified by BLASTing the sequence assigned to this density by ModelAngelo^20^. An initial model for TXNL1–proteasome complex was generated primarily by rigid body fitting individual AlphaFold models^31^ of each proteasomal subunit and of TXNL1, in addition to cross-referencing the output from ModelAngelo. The model was manually adjusted in Coot^32^ v.0.9.8 in between iterative rounds of real space refinement in Phenix^33^ v.1.19.2. Figures were made using ChimeraX^34^ v.1.5–v.1.7.

### Expression plasmids

Human TXNL1 cDNA (complementary DNA) oligonucleotide, flanked with attB1 or attB2 sites, was synthesized by Integrated DNA Technologies (IDT) for Gateway cloning into pDONR221 via the BP reaction (Thermo Fisher Scientific, cat. no. 11789020). Mutations to the TXNL1 entry clone were introduced using the Q5 Site-Directed Mutagenesis kit (NEB, cat. no. E0554S). The PCR primers were designed using the NEBaseChanger program and synthesized by IDT. WT and mutant versions of TXNL1 were subcloned into a pHAGE CMV 2xFLAG-N destination vector for N-terminal tagging or a pHAGE CMV destination vector conferring puromycin resistance^13^ via LR reactions (Thermo Fisher Scientific, cat. no. 11791100).

The tail-extended TXNL1 variant appended the following C-terminal residues of PITHD1—ASANPADHRVHQVTPQTHFIS. The tail-replaced TXNL1 variant exchanged the TXNL1 tail sequence (VQATNMNDFKRVVGKKGESH) with the entire C-terminal tail of PITHD1—LRRHEVTICNYEASANPADHRVHQVTPQTHFIS.

HA-TXNL1 (WT, D162R, SxxS) were codon-optimized for expression in bacteria and synthesized by IDT to contain overhangs for Gibson assembly (NEB, cat. no. E2611S). A pET 6xHis-3C bacterial expression vector was obtained from the Dana-Farber Cancer Institute Crystallography core facility and was digested with BamHI and NotI before Gibson assembly of the synthetic oligonucleotides.

### Cell culture and cell line generation

HEK 293T cells (ATCC, CRL-3216, RRID:CVCL_0063) were incubated at 37 °C and 5% CO_2_ in DMEM (Thermo Fisher Scientific, cat. no. 11965118) supplemented with 100 units ml^−1^ of penicillin, 0.1 mg ml^−1^ of streptomycin (Thermo Fisher Scientific, cat. no. 15070063) and 10% fetal bovine serum (Cytiva, cat. no. SH30088.03). Cells were treated with 10 µM MG132 (Selleckchem, cat. no. S2619), 1 µM TAK-243 (MedChemExpress, cat. no. HY-100487), 100 µM arsenite (Sigma, cat. no. S7400), 5 µM auranofin (MedChemExpress, cat. no. HY-B1123) or 250 µM hydrogen peroxide (Sigma, cat. no. 216763) from stock solution in DMSO or water.

To knockout TXNL1, WT HEK 293T cells were transfected using PolyJet (SignaGen, cat. no. SL100688) with a lentiCRISPRv2 plasmid expressing a blue-fluorescent protein (BFP) and the guide RNA targeting TXNL1 (GAATGACCCTGGAAGCAATG). Five days post-transfection, BFP-positive cells were single-cell sorted into 96-well plates and grown out for 2 weeks. Isogenic clones were expanded and screened for TXNL1 KO.

To generate lentivirus, HEK 293T cells were cultured in six-well plates to 90% confluency. Using PolyJet, the cells were transfected with 1 µg of total plasmid DNA encoding Tat, Rev, Gag-Pol and VSV-G (mixed equimolar) and a lentiviral transfer vector. The media was replaced 1-day after transfection. The lentiviral supernatant was collected 2 days post-transfection, passed through a 0.45 µm filter, and applied directly onto cells.

### Antibodies

The following primary antibodies were used for immunoblotting, all at a 1:1,000 dilution: rabbit anti-PSMD14 (Cell Signaling Technology (CST), cat. no. 4197S, RRID:AB_11178935), rabbit anti-PSMD4 (CST, cat. no. 12441S, RRID:AB_2797916), mouse anti-PSMD1 (Santa Cruz, cat. no. sc-166038, RRID:AB_2172797), rabbit anti-PSMD2 (CST, cat. no. 25430, RRID:AB_2798903), rabbit anti-PSMA2 (CST, cat. no. 2455, RRID:AB_2171400), rabbit anti-PSMB5 (CST, cat. no. 12919, RRID:AB_2798061), rabbit anti-FLAG (CST, cat. no. 14793, RRID:AB_2572291), rabbit anti-HA (CST, cat. no. 3724S, RRID:AB_1549585), rabbit anti-mTOR (CST, cat. no. 2983, RRID:AB_2105622), rabbit anti-TXNL1 (Abcam, cat. no. ab188328, RRID:AB_2687563) and rabbit anti-ubiquitin (CST, cat. no. 43124S, RRID:AB_2799235). Secondary antibodies used at a 1:2,000 dilution were: anti-rabbit immunoglobulin G, horseradish peroxidase-linked (CST, cat. no. 7074, RRID:AB_2099233) or anti-mouse immunoglobulin G, horseradish peroxidase-linked (CST, cat. no. 7076S, RRID:AB_330924).

### Sucrose gradient ultracentrifugation

About 2 million HEK 293T cells were lysed in 50 µl of lysis buffer containing 0.5% CHAPS, 100 mM NaCl, 40 mM HEPES pH 7.4 and 1× protease-phosphatase inhibitor cocktail. Twenty microliters of clarified lysate supernatant was loaded onto a gradient containing five 40-µl steps of 30%, 25%, 20%, 15% and 10% sucrose in 100 mM NaCl and 40 mM HEPES pH 7.4 and subjected to ultracentrifugation at 55,000 rpm (258,719*g*) for 1 h using a TLS55 rotor. After centrifugation, 20-µl aliquots were diluted in Tris-Glycine sample buffer containing 10% 2-mercaptoethanol.

### Immunoprecipitations from cells

HEK 293T cells were seeded at 1 million cells in 10-cm culture dishes and transfected with 3 µg of DNA encoding 2xFLAG-TXNL1 variants using PolyJet 2 days post-seeding. The media was replaced with fresh media 1 day post-transfection and incubated overnight. The cells were rinsed once with ice-cold PBS and 700 µl of lysis buffer containing 0.5% CHAPS, 100 mM NaCl, 40 mM HEPES pH 7.4, 1× protease-phosphatase inhibitor cocktail (Thermo Fisher Scientific, cat. no. 78441) was applied directly to the dish. The cells were collected in Eppendorf tubes by scraping, and were incubated for 15 min at 4 °C with end-to-end rotation. The lysates were then centrifuged at 21,000*g* for 15 min at 4 °C. While centrifuging, 15 µl per plate of anti-FLAG magnetic beads (Sigma, cat. no. M8823, RRID:AB_2637089) were rinsed three times with the same lysis buffer. After centrifuging, 30 µl of lysate was diluted into 200 µl of Tris-Glycine SDS sample buffer (Thermo Fisher Scientific, cat. no. LC2676) supplemented with 10% 2-mercaptoethanol. The remaining supernatant was applied directly to the anti-FLAG beads and incubated end-to-end for 1 h at 4 °C. After incubation, the beads were rinsed three times with 700 µl of lysis buffer and resuspended in 50 µl of sample buffer. Proteins were eluted from the beads and denatured for 3 min at 95 °C. An aliquot of immunoprecipitate (IP) sample was diluted 1:20 in sample buffer to blot for the abundant bait protein. To perform immunoblotting, 15 µl of lysate, diluted IP or total IP was loaded into 15-well gels.

### Purification of recombinant HA-TXNL1

Rosetta (DE3) cells (Millipore, cat. no. 71397-3) were transformed with pET plasmids encoding 6xHis-3C-HA-TXNL1 (WT, D162R, SxxS) and grown overnight at 37 °C on plates containing kanamycin + chloramphenicol. Single colonies were then grown out in 50 ml of LB medium containing kanamycin + chloramphenicol overnight at 37 °C with 200 rpm shaking. The following day, the LB containing bacteria was transferred to 1 liter of fresh LB solution containing kanamycin + chloramphenicol and grown at 37 °C with 200 rpm shaking to an optical density of 0.6. The bacteria were kept at 4 °C for 10 min before supplementing the LB medium with 0.5 mM IPTG (Sigma, cat. no. I5502-10G) to induce TXNL1 expression. Cultures were incubated at 16 °C for 16 h with 200 rpm shaking.

The following day, the liquid cultures were transferred to 1-liter centrifuge bottles, and bacteria were pelleted by centrifuging at 4,000*g* for 15 min at 4 °C. The pellets were resuspended in 35 ml of buffer containing 50 mM HEPES pH 7.4, 150 mM NaCl and 10 mM imidazole (Sigma, cat. no. 68268-500ML-F). The bacteria were again pelleted by centrifugation and the supernatant was discarded. The bacteria were then resuspended in 35 ml of lysis buffer containing 50 mM HEPES pH 7.4, 150 mM NaCl, 10 mM imidazole, 1 mM DTT (Thermo Fisher Scientific, cat. no. R0862), benzonase (Millipore, cat. no. 71206-3) and protease inhibitor tablets (Sigma, cat. no. 11873580001). A microfluidizer (700 bar, 4 cycles, 20 ml min^−1^ flow rate) was then used to lyse cells. The lysate was centrifuged at 21,000*g* for 20 min at 4 °C. During the centrifugation, 4.8 ml of Ni-NTA agarose (Qiagen, cat. no. 30210) was rinsed three times using lysis buffer. Post-centrifugation, the supernatant was incubated with the washed Ni-NTA resin in 50-ml conical tubes for 30 min at 4 °C with end-to-end rotation. The solution was then passed through a column (Bio-Rad, cat. no. 7321010) to remove the liquid by gravity. The conical tubes and column were rinsed with 50 ml total volume of lysis buffer by gravity flow at 4 °C. Once rinsed, the proteins were eluted from the resin by sequentially incubating the resin with 500 µl of elution buffer containing 50 mM HEPES, 150 mM NaCl and 200 mM imidazole four times for 5 min at room temperature. Using a NanoDrop, the elutions were measured for absorbance at 280 nm to determine whether the protein eluted from the resin.

Elutions containing proteins were pooled and concentrated using an Amicon Ultra-15, 3 kDa MWCO (Millipore, cat. no. UFC9003). The concentrated sample was centrifuged for 1 min at 21,000*g* to remove large particles. A Superdex 200 was equilibrated using a buffer containing 50 mM HEPES, 150 mM NaCl and 1 mM DTT, and the concentrated sample was injected into a 0.5-ml injection loop. Fractions corresponding to TXNL1 were collected, pooled and concentrated using an Amicon Ultra-15, 3 kDa MWCO. Protein samples were flash-frozen using liquid nitrogen for storage at −80 °C.

### Purification of TXNL1-deficient proteasomes

*TXNL1* KO HEK 293T cells were seeded in fifty 15-cm plates at 3.5 million cells per plate and grown for 2 days. Cells were then transfected with 10 µg of plasmid DNA encoding 2xFLAG-midnolin per plate using PolyJet. The medium was replaced 1 day post-transfection and cells were incubated overnight. The cells were then rinsed once with ice-cold PBS before applying 1 ml of buffer containing 40 mM HEPES, 100 mM NaCl, 2 mM MgCl_2_, 5 mM ATP, 1× HALT protease-phosphatase inhibitor and 0.5% CHAPS to each 15-cm plate. The cells were collected by scraping and the lysate was incubated at 4 °C with end-to-end rotation for 20 min. The lysate was then centrifuged at 21,000*g* for 20 min at 4 °C. During centrifugation, 3.6 ml of anti-FLAG M2 agarose resin (Millipore, cat. no. A2220) was rinsed four times using lysis buffer. After centrifugation, the supernatant was incubated in a 50-ml conical tube containing the washed anti-FLAG resin with end-to-end rotation for 1.5 h at 4 °C. The solution was then passed through a column (Bio-Rad, cat. no. 7321010) by gravity at 4 °C. The conical tube and the column were rinsed with 50 ml of lysis buffer total by gravity flow at 4 °C. The column was also rinsed once with 20 ml of SEC buffer containing 40 mM HEPES, 100 mM NaCl, 2 mM MgCl, 5 mM ATP, 0.05% CHAPS at 4 °C.

The resin was then incubated with 1.8 ml of SEC buffer containing 0.5 mg ml^−1^ of 3xFLAG peptide (APExBIO, cat. no. A6001) for 20 min at room temperature. A total of four elutions were performed sequentially and the protein content in each elution was determined by obtaining the absorbance at 280 nm using a NanoDrop. The eluted proteins were pooled and concentrated to 500 µl using an Amicon Ultra-15, 10 kDa MWCO concentrator. The concentrated sample was centrifuged for 1 min at 21,000*g* to remove large particles. A Superose6 column was equilibrated using SEC buffer for SEC and the sample was manually injected into a 0.5-ml injection loop. Fractions corresponding to the proteasome were collected, pooled and concentrated using Amicon Ultra-15, 10 kDa MWCO. Protein samples were flash-frozen using liquid nitrogen for storage at −80 °C.

### Binding and degradation assays with purified components

To test TXNL1 binding to proteasomes in vitro, 1 µl of each TXNL1 protein (1.7 mg ml^−1^) was diluted in 199 µl of sample buffer, and 1 µl of proteasomes (3 mg ml^−1^) was diluted in 99 µl of sample buffer as input. Ten microliters of anti-HA beads (Thermo Fisher Scientific, cat. no. 88836, RRID:AB_2749815) per reaction were washed three times using 700 µl of CHAPS buffer (100 mM NaCl, 40 mM HEPES pH 7.4, 0.5 % CHAPS, 1× HALT protease-phosphatase inhibitor). After washing, the beads were resuspended in 700 µl of CHAPS buffer containing 4% BSA (40 mg ml^−1^). The beads were blocked for 1 h at 4 °C with end-to-end rotation. After blocking, the beads were washed three times using 700 µl of CHAPS buffer. The beads were resuspended in CHAPS buffer and 10 µl of beads were aliquoted into pre-lubricated Eppendorf tubes (Millipore, cat. no. CLS3207). HA-TXNL1 protein (15 µg per reaction) was added directly to the beads to a final volume of 500 µl in the CHAPS buffer. The beads were incubated for 1 h at 4 °C with end-to-end rotation to immobilize the TXNL1. The beads were then washed three times using 700 µl of CHAPS buffer and resuspended to 500 µl in CHAPS buffer. Four microliters of proteasomes (3 mg ml^−1^) were added to each reaction. For the chelator experiment, proteasomes were preincubated with 6 mM 1,10-phenanthroline (Sigma, cat. no. 516705) or 1,7-phenanthroline (Sigma, cat. no. 301841) for 30 min at room temperature before adding the mixture to the TXNL1-bound beads. The beads were incubated with end-to-end rotation for 1 h at 4 °C, and then washed three times using 700 µl of CHAPS buffer before being resuspended in 25 µl of Tris-Glycine sample buffer containing 10% 2-mercaptoethanol. Proteins were eluted from the beads by denaturing at 95 °C for 3 min.

To assay for protein degradation, 50 nM purified proteasomes were preincubated at room temperature for 30 min with a negative control buffer, 6 mM 1,10-phenanthroline, or 50 µM TXNL1 variants in 100 mM NaCl and 40 mM HEPES pH 7. Then, 50 nM poly-ubiquitinated substrate was added directly to the proteasome mixture along with 5 mM ATP (Sigma, cat. no. A2383) in a 10-µl reaction volume, incubated for 10 min at 37 °C, and quenched with 10 µl of Tris-Glycine sample buffer containing 10% 2-mercaptoethanol. Proteins were denatured at 95 °C for 3 min and 8 µl of sample was loaded into 4%–20% Tris-Glycine 15-well pre-cast gels (Thermo Fisher Scientific, cat. no. XP04205BOX). In-gel fluorescent images were acquired using an Odyssey LI-COR.

### Multiplexed mass spectrometry

Cells were washed twice with 1× PBS, harvested on ice using a cell scraper in 1× PBS, pelleted via centrifugation for 5 min at 1,000*g* at 4 °C, and washed with 1× PBS before resuspension in in 8 M urea (Sigma, cat. no. U5378), 50 mM NaCl, 50 mM EPPS (Sigma, cat. no. E9502) and 1× Protease Inhibitor Cocktail (Roche, cat. no. 4906845001). After 10 s of sonication, lysed cells were pelleted, and the protein concentration of the clarified sample was determined using a BCA kit (Thermo Fisher Scientific, cat. no. 23225). Then, 100 µg of each sample was incubated for 30 min at 37 °C with 5 mM TCEP (Gold Biotechnology) for disulfide bond reduction with subsequent alkylation with 20 mM iodoacetamide (Sigma, cat. no. I6125) for 20 min at room temperature followed by quenching with 15 mM DTT for 15 min under gentle shaking. MeOH-chloroform precipitation of samples was performed as follows: to each sample, four parts MeOH was added followed by vortexing, one part chloroform was added followed by vortexing, and finally three parts H_2_O was added. After vortexing, the suspension was centrifugated for 5 min at 14,000*g* and the aqueous phase around the protein precipitate was removed using a loading tip. The precipitate was washed twice with MeOH and resuspended in 200 mM EPPS (pH 8.0) (Sigma, cat. no. E9502) and digested with Trypsin/Lys-C (Promega, cat. no. V5073) digestion (1:100) at 37 °C overnight with gentle shaking.

One hundred and fifty microliters of digested samples were labeled by adding 10 µl of TMT reagent (Thermo Scientific, cat. no. A52045; stock: 20 mg ml^−1^ in acetonitrile (ACN), Millipore Sigma, cat. no. 34851) together with 50 µl of ACN to yield a final ACN concentration of approximately 25% (v/v) for 1 h at room temperature before quenching the reaction with hydroxylamine (Thermo Fisher Scientific, cat. no. 90115) to a final concentration of 0.2% (v/v). The TMTpro-labeled samples were pooled at a 1:1 ratio, resulting in a consistent peptide amount across all channels. Pooled samples were vacuum centrifuged for 1 h at room temperature to remove ACN, followed by reconstitution in 1% formic acid (FA), desalting using C18 solid-phase extraction (200 mg, Sep-Pak, Waters, cat. no. WAT054960) and vacuum centrifugation until near dryness. We fractionated the pooled, labeled peptide sample using basic pH reversed-phase HPLC^35^ and an Agilent 1260 pump equipped with a degasser and a UV detector (wavelength set at 220 and 280 nm). Peptides were subjected to a 50-min linear gradient from 5% to 35% ACN in 10 mM ammonium bicarbonate pH 8 at a flow rate of 0.6 ml min^−1^ over an Agilent 300Extend C18 column (3.5 μm particles, 4.6 mm internal diameter and 220 mm in length). The peptide mixture was fractionated into 96 fractions, which were consolidated into 24 super-fractions^36^, of which 12 nonadjacent fractions were analyzed. Samples were subsequently acidified with 1% FA (Sigma, cat. no. 94318) and vacuum centrifuged to near dryness. Each super-fraction was desalted via StageTip, dried again via vacuum centrifugation and reconstituted in 10 µl 5% ACN, 5% FA for LC–MS/MS processing.

Mass spectrometric data were collected on Orbitrap Fusion Lumos instruments coupled to a Proxeon NanoLC-1200 UHPLC. The 100-µm capillary column was packed with 35 cm of Accucore 150 resin (2.6 μm, 150 Å; Thermo Fisher Scientific) at a flow rate of 340 nl min^−1^. The scan sequence began with an MS1 spectrum (Orbitrap analysis, resolution 60,000, mass range 350–1,350 Th, automatic gain control target 100%, maximum injection time 118 ms). Data were acquired at ~90 min per fraction. The hrMS2 stage consisted of fragmentation by higher energy collisional dissociation (normalized collision energy 36%) and analysis using the Orbitrap (automatic gain control 200%, maximum injection time 120 ms, isolation window 0.6 Th, resolution 50,000). Data were acquired using the FAIMSpro interface with the dispersion voltage set to 5,000 V, the compensation voltages set at −40, −60 and −80 V, and the TopSpeed parameter set at 1 s per compensation voltage.

Spectra were converted to mzXML via MSconvert^37^. Database searching included all entries from the mouse UniProt reference database (downloaded August 2021). The database was concatenated with one composed of all protein sequences for that database in the reversed order. Searches were performed using a 50-ppm precursor ion tolerance for total protein-level profiling. The product ion tolerance was set to 0.03 Da. These wide mass tolerance windows were chosen to maximize sensitivity in conjunction with Comet searches and linear discriminant analysis^38,39^. TMTpro labels on lysine residues and peptide N termini (+304.207 Da), as well as carbamidomethylation of cysteine residues (+57.021 Da) were set as static modifications, while oxidation of methionine residues (+15.995 Da) was set as a variable modification. Peptide–spectrum matches (PSMs) were adjusted to a 1% false discovery rate^40,41^. PSM filtering was performed using a linear discriminant analysis, as described previously^39^ and then assembled further to a final protein-level false discovery rate of 1% (ref. ^40^). Proteins were quantified by summing reporter ion counts across all matching PSMs, also as described previously^42^. Reporter ion intensities were adjusted to correct for the isotopic impurities of the different TMTpro reagents according to manufacturer specifications. The signal-to-noise measurements of peptides assigned to each protein were summed and these values were normalized so that the sum of the signal for all proteins in each channel was equivalent to account for equal protein loading. Finally, each protein abundance measurement was scaled, such that the summed signal-to-noise for that protein across all channels equals 100, thereby generating a relative abundance measurement.

MSstatsTMT^43^ was performed on peptides with >200 summed signal-to-noise ratios across TMT channels. For each protein, the filtered PSM TMTpro raw intensities were summed and log_2_ normalized to calculate protein quantification values (weighted average) and normalized to total TMT channel intensity across all quantified PSMs (adjusted to median total TMT intensity for the TMT channels)^44^. The log_2_ normalized summed protein reporter intensities were compared using a Student’s *t*-test and *P* values were corrected for multiple hypotheses using the Benjamini–Hochberg adjustment^45^.

### RNA sequencing

HEK 293T cells were grown to 80% confluency in six-well plates, treated with oxidizing agents (100 µM arsenite or 600 µM hydrogen peroxide) in triplicate for 6 h, and total RNA was isolated using an RNEasy Plus mini kit (Qiagen, cat. no. 74134). Messenger RNA sequencing libraries were prepared by Innomics and sequenced on a DNBseq platform to obtain 100-bp paired-end reads. Reads were aligned to the GRCh38 primary assembly obtained from gencodegenes.org using qAlign from the QuasR package. Raw counts were filtered to discard transcripts with fewer than ten total reads in the three untreated *TXNL1* KO replicates, and then the DESeq2 package in R was used to obtain differential expression data and statistics.

### Reporting summary

Further information on research design is available in the Nature Portfolio Reporting Summary linked to this article.

## Online content

Any methods, additional references, Nature Portfolio reporting summaries, source data, extended data, supplementary information, acknowledgements, peer review information; details of author contributions and competing interests; and statements of data and code availability are available at 10.1038/s41594-025-01639-w.

## Supplementary information

Reporting Summary

Supplementary Table 1 TMT-MS of untreated and arsenite-treated TXNL1 KO HEK 293T cells complemented without or with wild-type or R162D R234D TXNL1.

Supplementary Table 2 RNA sequencing of untreated, arsenite-treated, or hydrogen peroxide-treated TXNL1 KO HEK 293T cells complemented without or with wild-type, D162R R234D (Prot) or SxxS (Thio) TXNL1.

## Source data

Source data Unprocessed western blots and gels for Fig. 2 and Extended Data Figs. 3, 5, 6 and 7.

## Data Availability

EM maps and models are available under accession numbers EMD-44949, EMD-44952 and PDB 9BW4. The mass spectrometry proteomics data are available via ProteomeXchange with identifier PXD052933. RNA sequencing data are available through GEO under the accession number GSE271951. Genome assembly GRCh38 (GCF_000001405.26) and the UniProt reference database (downloaded August 2021) were used for data analysis. All other data are included in the paper and its Supplementary Information. Source data are provided with this paper.

## Code availability

No new code was generated in this study.
