# Supplementary material for: Structure of the TXNL1-bound proteasome
Source: Nat Struct Mol Biol. 2025 Aug 6;32(12):2398–402. doi: 10.1038/s41594-025-01639-w (PMC12700798; doi:10.1038/s41594-025-01639-w)
Supplement: Supplementary file 1 — Reporting Summary [file 41594_2025_1639_MOESM1_ESM.pdf]

# nature portfolio

Corresponding author(s): Elledge, Shao

Last updated by author(s): May 29, 2025

# Reporting Summary

Nature Portfolio wishes to improve the reproducibility of the work that we publish. This form provides structure for consistency and transparency in reporting. For further information on Nature Portfolio policies, see our Editorial Policies and the Editorial Policy Checklist.

## Statistics

For all statistical analyses, confirm that the following items are present in the figure legend, table legend, main text, or Methods section.

| n/a | Confirmed | |
|---|---|---|
| ☐ | ☒ | The exact sample size (*n*) for each experimental group/condition, given as a discrete number and unit of measurement |
| ☐ | ☒ | A statement on whether measurements were taken from distinct samples or whether the same sample was measured repeatedly |
| ☐ | ☒ | The statistical test(s) used AND whether they are one- or two-sided<br>*Only common tests should be described solely by name; describe more complex techniques in the Methods section.* |
| ☐ | ☒ | A description of all covariates tested |
| ☐ | ☒ | A description of any assumptions or corrections, such as tests of normality and adjustment for multiple comparisons |
| ☐ | ☒ | A full description of the statistical parameters including central tendency (e.g. means) or other basic estimates (e.g. regression coefficient) AND variation (e.g. standard deviation) or associated estimates of uncertainty (e.g. confidence intervals) |
| ☒ | ☐ | For null hypothesis testing, the test statistic (e.g. *F*, *t*, *r*) with confidence intervals, effect sizes, degrees of freedom and *P* value noted<br>*Give P values as exact values whenever suitable.* |
| ☒ | ☐ | For Bayesian analysis, information on the choice of priors and Markov chain Monte Carlo settings |
| ☒ | ☐ | For hierarchical and complex designs, identification of the appropriate level for tests and full reporting of outcomes |
| ☒ | ☐ | Estimates of effect sizes (e.g. Cohen's *d*, Pearson's *r*), indicating how they were calculated |

*Our web collection on statistics for biologists contains articles on many of the points above.*

## Software and code

Policy information about availability of computer code

| | |
|---|---|
| Data collection | SerialEM v4.0.5 |
| Data analysis | cryoSPARC v4.3.1, ChimeraX v1.5 - v1.7, ModelAngelo v1.0Phenix v1.19.2, Coot v0.9.8, Alphafold2, Alphafold3, MSconvert, MSstatsTMT, qAlign (QuasR), DESeq2 |

For manuscripts utilizing custom algorithms or software that are central to the research but not yet described in published literature, software must be made available to editors and reviewers. We strongly encourage code deposition in a community repository (e.g. GitHub). See the Nature Portfolio guidelines for submitting code & software for further information.

## Data

Policy information about availability of data

All manuscripts must include a data availability statement. This statement should provide the following information, where applicable:

- Accession codes, unique identifiers, or web links for publicly available datasets
- A description of any restrictions on data availability
- For clinical datasets or third party data, please ensure that the statement adheres to our policy

EM maps and models are available under accession numbers EMD-44949, EMD-44952, and PDB 9BW4. The mass spectrometry proteomics data are available via ProteomeXchange with identifier PXD052933. RNA sequencing data are available through GEO under the accession number GSE271951. Genome assembly GRCh38

(GCF_000001405.26) and the Uniprot reference database (downloaded August 2021) were used for data analysis. All other data are included in the manuscript and its supplemental information.

## Research involving human participants, their data, or biological material

Policy information about studies with human participants or human data. See also policy information about sex, gender (identity/presentation), and sexual orientation and race, ethnicity and racism.

| | |
|---|---|
| Reporting on sex and gender | N/A |
| Reporting on race, ethnicity, or other socially relevant groupings | N/A |
| Population characteristics | N/A |
| Recruitment | N/A |
| Ethics oversight | N/A |

Note that full information on the approval of the study protocol must also be provided in the manuscript.

# Field-specific reporting

Please select the one below that is the best fit for your research. If you are not sure, read the appropriate sections before making your selection.

☒ Life sciences ☐ Behavioural & social sciences ☐ Ecological, evolutionary & environmental sciences

For a reference copy of the document with all sections, see nature.com/documents/nr-reporting-summary-flat.pdf

# Life sciences study design

All studies must disclose on these points even when the disclosure is negative.

| | |
|---|---|
| Sample size | No statistical methods involving predetermined sample sizes were used. Triplicates were analyzed for each conditions for proteomics and RNA sequencing analyses according to standard practice. |
| Data exclusions | Established single particle image processing algorithms may exclude or weight particles. For RNA sequencing data, transcript with fewer than 10 total reads in the three TXNL1 knockout cellular lysate replicates were filtered out before further analysis. No other data were excluded from analyses. |
| Replication | At least two independent replicates were performed for all biochemical and cellular experiments. All results shown in the study were successfully replicated. Triplicates were analyzed for each condition for proteomics and RNA sequencing analyses. |
| Randomization | Established single particle imaging processing algorithms randomly split cryo-EM particle images into two halves during refinement. Randomization is not required for other experiments in the study because the independent variable(s) are well-defined and controlled in the proteomics and RNA sequencing conditions. |
| Blinding | Blinding is not applicable to the methods reported because independent variable(s) are well-defined and controlled for within each experimental setup. |

# Reporting for specific materials, systems and methods

We require information from authors about some types of materials, experimental systems and methods used in many studies. Here, indicate whether each material, system or method listed is relevant to your study. If you are not sure if a list item applies to your research, read the appropriate section before selecting a response.

## Materials & experimental systems

| n/a | Involved in the study |
| --- | --- |
| ☐ | ☒ Antibodies |
| ☐ | ☒ Eukaryotic cell lines |
| ☒ | ☐ Palaeontology and archaeology |
| ☒ | ☐ Animals and other organisms |
| ☒ | ☐ Clinical data |
| ☒ | ☐ Dual use research of concern |
| ☒ | ☐ Plants |

## Methods

| n/a | Involved in the study |
| --- | --- |
| ☒ | ☐ ChIP-seq |
| ☒ | ☐ Flow cytometry |
| ☒ | ☐ MRI-based neuroimaging |

# Antibodies

Antibodies used

The following primary antibodies were used for immunoblotting, all at a 1:1000 dilution: rabbit anti-PSMD14 (CST, 4197S, RRID: AB_11178935), rabbit anti-PSMD4 (CST, 12441S, RRID: AB_2797916), mouse anti-PSMD1 (Santa Cruz, sc-166038, RRID: AB_2172797), rabbit anti-PSMD2 (CST, 25430, RRID: AB_2798903), rabbit anti-PSMA2 (CST, 2455, RRID: AB_2171400), rabbit anti-PSMB5 (CST, 12919, RRID: AB_2798061), rabbit anti-FLAG (CST, 14793, RRID: AB_2572291), rabbit anti-HA (CST, 3724S, RRID: AB_1549585), rabbit anti-mTOR (CST, 2983, RRID: AB_2105622), rabbit anti-TXNL1 (Abcam, ab188328, RRID: AB_2687563), and rabbit anti-Ubiquitin (CST, 43124S, RRID: AB_2799235). Secondary antibodies used at a 1:2000 dilution were: anti-rabbit IgG, HRP-linked (CST, 7074, RRID: AB_2099233) or anti-mouse IgG, HRP-linked (CST, 7076S, RRID: AB_330924).

Validation

Antibodies against proteasomal subunits (PSMD14 - https://www.cellsignal.com/products/primary-antibodies/psmd14-d18c7-rabbit-mab/4197, PSMD4 - https://www.cellsignal.com/products/primary-antibodies/s5a-psmd4-d20b2-rabbit-mab/12441, PSMD1 - https://www.scbt.com/p/psmd1-antibody-c-7, PSMD2 - https://www.cellsignal.com/products/primary-antibodies/psmd2-d6w7g-rabbit-mab/25430, PSMA2 - https://www.cellsignal.com/products/primary-antibodies/psma2-antibody/2455, and PSMB5 - https://www.cellsignal.com/products/primary-antibodies/psmb5-d1h6b-rabbit-mab/12919) were all validated by the manufacturer of each antibody to detect an endogenous protein of the expected molecular weight in immunoblotting experiments of lysates from multiple human cell lines. Each of the proteasomal protein antibodies was additionally validated in our study through immunoblotting of size fractionations of human lysates to show that the detected protein displayed the expected molecular weight and exhibited native molecular weights consistent with proteasomes, and that each primary antibody only cross reacted with the appropriate species-specific secondary antibodies. Antibodies against the HA (https://www.cellsignal.com/products/primary-antibodies/ha-tag-c29f4-rabbit-mab/3724) and FLAG (https://www.cellsignal.com/products/primary-antibodies/dykddddk-tag-d6w5b-rabbit-mab-binds-to-same-epitope-as-sigma-aldrich-anti-flag-m2-antibody/14793) epitopes were validated by the manufacturers and in our study by immunoblotting lysates of cells that either express or do not express tagged proteins and confirmed specifically detect only the appropriately tagged proteins of the expected molecular weights and to cross react with anti-rabbit but not anti-mouse secondary antibodies. The mTor antibody (https://www.cellsignal.com/products/primary-antibodies/mtor-7c10-rabbit-mab/2983) was validated by the manufacturer by immunoblotting to recognize a protein of the expected molecular weight in multiple human cell lysates as well as in our and prior studies (PMID: 37616343 via the same metric. The TXNL1 (https://www.abcam.com/en-us/products/primary-antibodies/txnl1-antibody-epr16061b-n-terminal-ab188328) antibody was validated by the manufacturer and in our study by the immunoblotting of lysates of multiple human cell lines after genetic knockouts and re-expression of untagged and tagged versions of the protein to detect a protein of the expected molecular weight and to cross react only with anti-rabbit secondary antibodies. The ubiquitin antibody (https://www.cellsignal.com/products/primary-antibodies/ubiquitin-e4i2j-rabbit-mab/43124) was validated by the manufacturer and this study to recognize polyubiquitin conjugates in multiple human cell lines, which decrease as expected when cells are treated with a ubiquitylation inhibitor and increase as expected when cells are treated with proteasome inhibitors.

# Eukaryotic cell lines

Policy information about cell lines and Sex and Gender in Research

Cell line source(s)

HEK-293T cells were obtained from ATCC (CRL-3216).

Authentication

No additional authentication procedures were performed.

Mycoplasma contamination

Cell lines were not tested for mycoplasma contamination.

Commonly misidentified lines
(See ICLAC register)

No commonly misidentified cell line was used in the study.

## Plants

| | |
|---|---|
| Seed stocks | N/A |
| Novel plant genotypes | N/A |
| Authentication | N/A |
