# Supplementary material for: Structure of the TXNL1-bound proteasome
Source: Nat Struct Mol Biol. 2025 Aug 6;32(12):2398–402. doi: 10.1038/s41594-025-01639-w (PMC12700798; doi:10.1038/s41594-025-01639-w)

Figure 2a

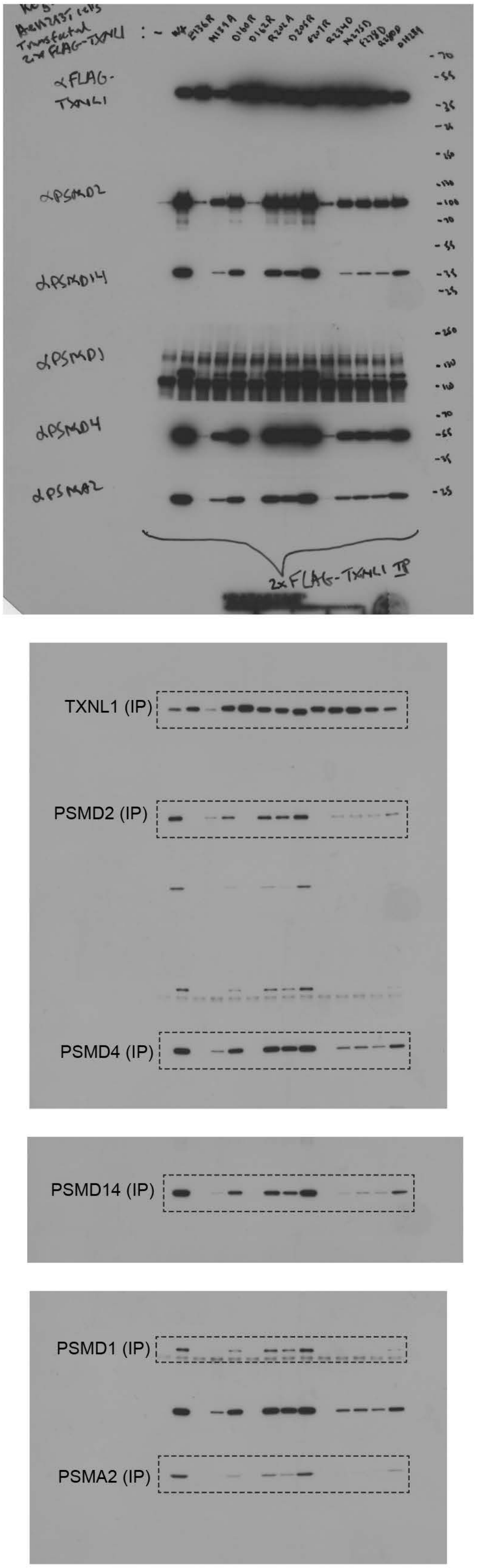

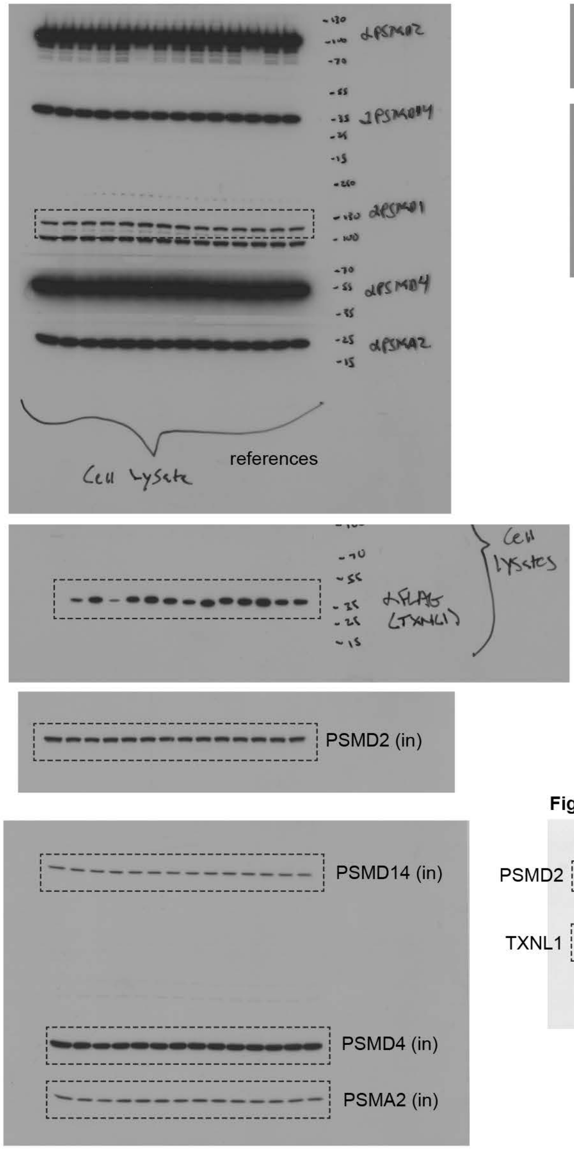

Figure 2c

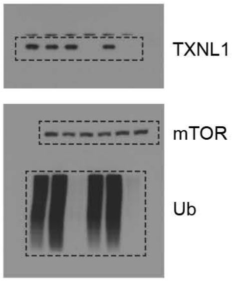

Figure 2d

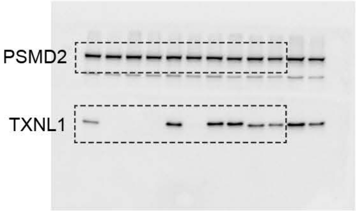

Figure 2b

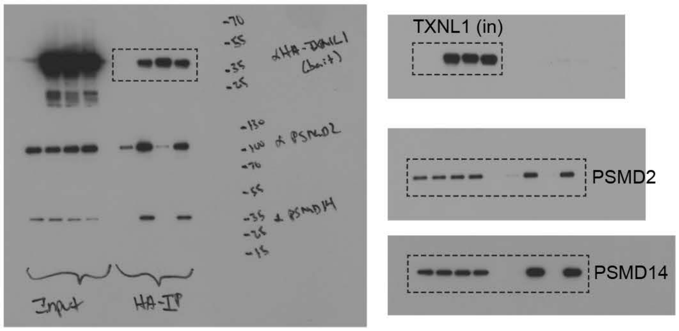

Extended Data Fig. 3b

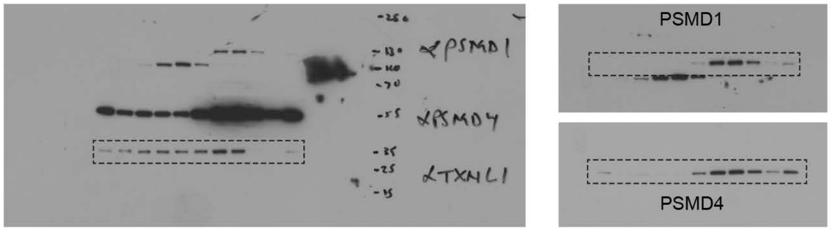

Extended Data Fig. 5c

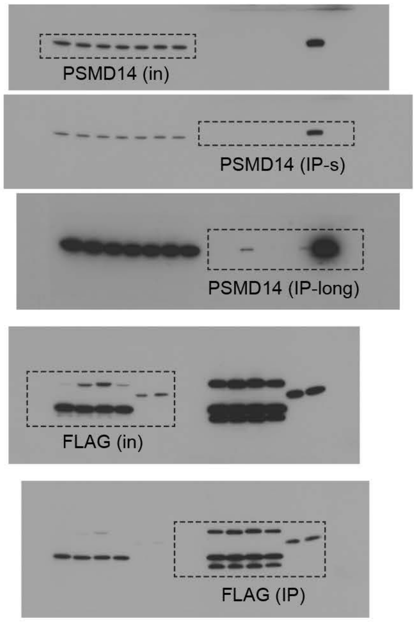

Extended Data Fig. 5e

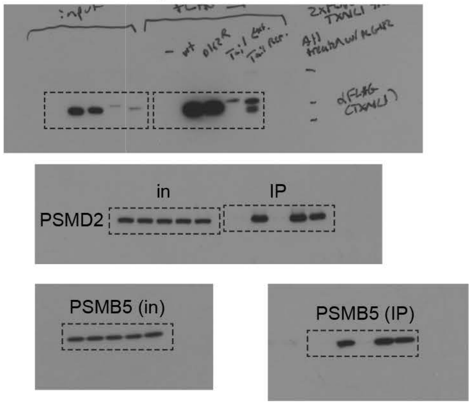

Extended Data Fig. 5f

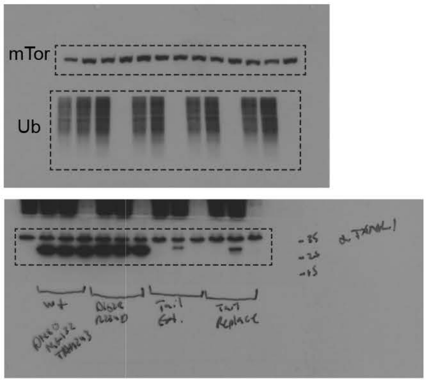

Extended Data Fig. 6a

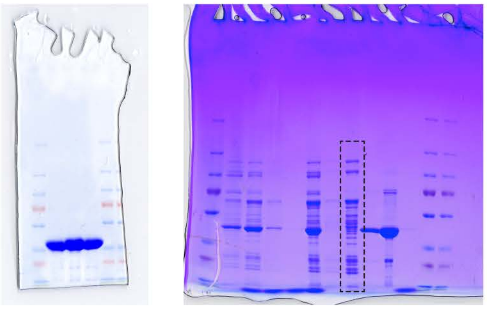

Extended Data Fig. 6c

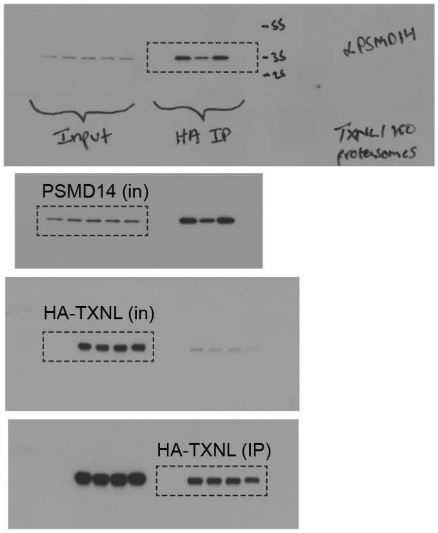

Extended Data Fig. 6d

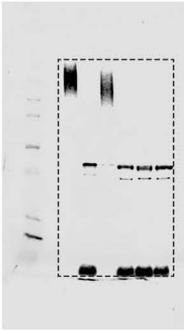

Extended Data Fig. 6b

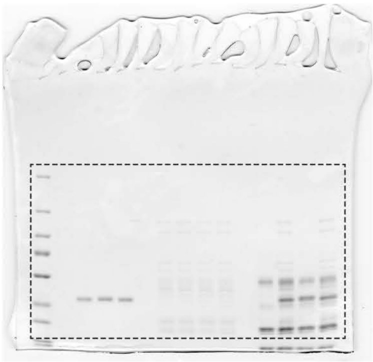

Extended Data Fig. 7a

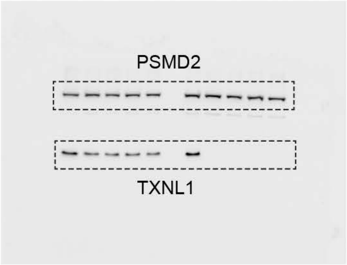

Extended Data Fig. 7b

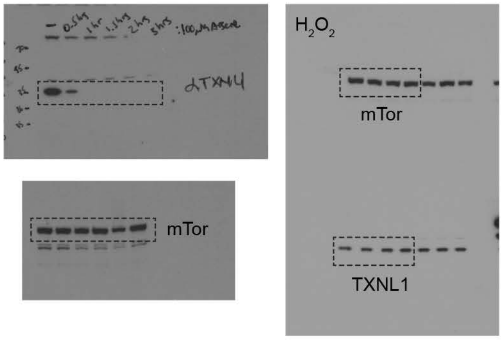

Extended Data Fig. 7c

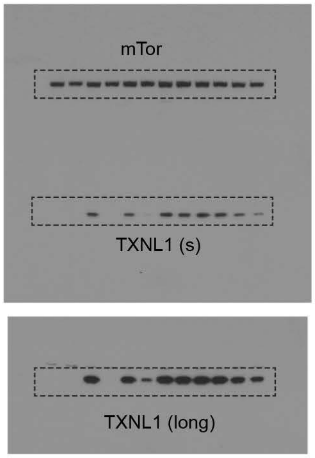

Extended Data Fig. 7d

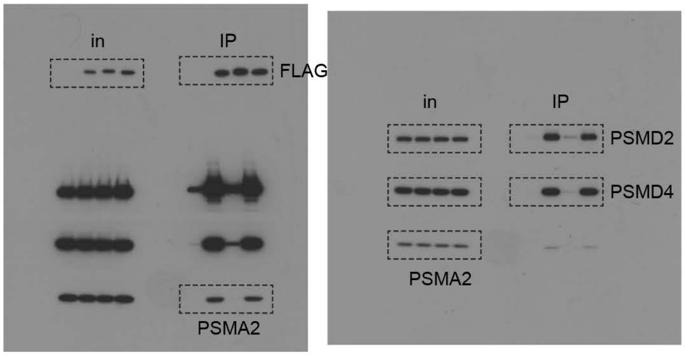

Extended Data Fig. 7e

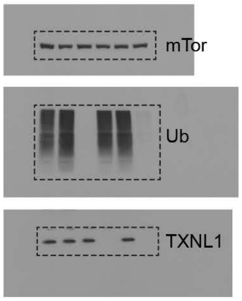

Extended Data Fig. 7f

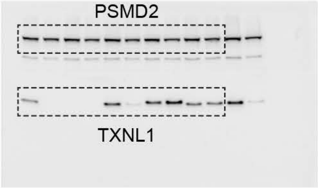

Supplement: Supplementary file 4 — Unprocessed western blots and gels for Fig. 2 and Extended Data Figs. 3, 5, 6 and 7. [file 41594_2025_1639_MOESM4_ESM.pdf]
